# Discharge preparation and readiness after birth: a scoping review of global policies, guidelines and literature

**DOI:** 10.1186/s12884-022-04577-3

**Published:** 2022-04-05

**Authors:** Helen Smith, Chloe Harvey, Anayda Portela

**Affiliations:** 1 International Health Consulting Services Ltd, Merseyside, UK; 2 Independent Consultant, Bangkok, Thailand; 3 Department of Maternal, Newborn, Child and Adolescent Health and Ageing, World Health Organization, Geneva, Switzerland

**Keywords:** Postnatal care, Hospital discharge, Discharge preparation, Discharge readiness, Maternal health, Newborn health

## Abstract

**Background:**

Despite the existence of global recommendations, postnatal care provided following childbirth is variable and often fails to address a woman’s concerns about herself and the parents’ concerns about their baby. Discharge from a facility after birth is a key moment to ensure the woman, parents and newborn receive support for the transition to care in the home. We mapped the current policies, guidance and literature on discharge preparation and readiness to identify key concepts and evidence and inform recommendations to be considered in a World Health Organization (WHO) guidance on postnatal care.

**Methods:**

We were guided by the Johanna Briggs Institute approach, and developed inclusion criteria based on existing defintions of discharge preparation and readiness, and criteria for discharge readiness compiled by international professional organisaitons. To identify guidelines and policies we searched websites and archives of guideline organisations, and contacted individuals and professional societies working on postnatal care. We searched 14 electronic databases to locate published research and other literature on discharge preparation and readiness. For documents that met the inclusion criteria we extracted key characteristics, summarised discharge readiness criteria and components and discharge preparation steps, and characterised interventions to improve discharge preparation.

**Results:**

The review provides a systematic map of criteria for discharge that are in use and the common steps healthcare providers take in preparing women and newborns for the transition home. The mapping also identified interventions used to strengthen discharge preparation, theories and models that conceptualise discharge preparation, scales for measuring discharge readiness and qualitative studies on the perspectives of women, men and healthcare providers on postnatal discharge.

**Conclusions:**

The findings highlight contrasts between the research literature and policy documents. They indicate potential gaps in current discharge policies, and point to the need for more comprehensive discharge assessment and education to better identify and meet the needs of women, parents/caregivers and families prior to discharge and identify those who may require additional support.

**Protocol registration details:**

The protocol for the review was registered with protocols.io on 23 November 2020: 10.17504/protocols.io.bpzymp7w

**Supplementary Information:**

The online version contains supplementary material available at 10.1186/s12884-022-04577-3.

## Background

Globally more women are choosing to give birth in a facility. An analysis of recent Demogrpahic and Health Surveys (DHS) data suggests most births in Africa and Asia now take place in facilities [1]. The care received in the facility immediatly after birth is important for the health of the woman and her baby and to facilitate support to the woman, parents/caregivers and family^1^ to transition from care in the facility to providing care in the home and parenthood [2]. Recent qualitative evidence shows what matters to women during this period is achieving positive motherhood as well as adapting to changed intimate and family relationships and (re)gaining health and wellbeing for themselves and their baby [3].

However, despite the existence of global recommendations on postnatal care (PNC) of mothers and newborns [4], care provided in the facility following childbirth is variable and often fails to address a woman’s concerns about herself and her baby. Women report leaving facilities without sufficient knowledge or skills to take care of themselves and their newborns [5–7]. Research conducted in the United States reveals women need more information regarding newborn care and post-birth physical and emotional changes and feel unprepared for the postnatal period [8]. Understanding women’s needs at this time is important for her health and the health of the newborn [7]. When a woman, parents or newborn are not ready, discharge can place the woman at risk of not being able to meet her own needs and can also place care of the newborn at risk [7]. Consequently, use of health services by women and parents unprepared for the postnatal period may increase, as a result of their or their newborn’s vulnerable health status [9, 10].

Concerns around preparation for discharge after birth emerged in the 1990s when health facilities in many countries began implementing earlier discharge for uncomplicated births [11, 12]. Current practice varies considerably. While WHO currently recommends that after a normal viaginal birth a women and baby without complications remain in the faciltiy for at least 24 h, [4] a recent analysis suggests wide variation between countries; length of stay in a facility after childbirth in many low- and middle-income countries is too short for women to receive adequate immediate postnatal care [13]. As length of facility stay has reduced, so has the opportunity to assess the physical condition of women and newborns and to understand the emotional and social needs of women, parents and families [9]. Shorter stay in a facility may also reduce the amount of time available for providers to effectively convey all the necessary information and skills to women, parents and carers prior to discharge [14]. In one study in Tanzania, providers admitted there is insufficient time or resources to provide the quality of postnatal education they would like to provide [15].

This scoping review was undertaken to inform recommendations to be considered in World Health Organization (WHO) guidance on postnatal care. One aspect of the guidance relates to readiness for women and their newborns to be discharged from a facility after birth and the steps the health providers should take to prepare a woman, her newborn and parents/carers for discharge. However not much is known about the current state of discharge preparation and readiness practices in order to make recommendations for global implementation. As yet there is no comprehensive map of the evidence base; very little is known about working definitions and conceptual boundaries, what criteria for discharge readiness are in use or what strategies or interventions exist for improving discharge preparation and readiness.

## Methods

We conducted this scoping review to map the range of available policies, guidance and literature on discharge preparation and readiness, in order to a) clarify definitions of discharge preparation and readiness, b) list criteria and items currently used to assess discharge readiness and c) identify and summarise characterisitcs of interventions implemented to improve discharge preparation. The methods guide has been registered on protcols.io [16]. We were guided by the Joanna Briggs Institute standard approach for scoping reviews [17].

### Inclusion criteria

There are no standard definitions of discharge preparaton and discharge readiness in the literature, however in relation to discharge of high-risk newborns a distinction is made between discharge readiness (the desired outcome), and discharge preparation (the process by which readiness is achieved) [18, 19]. In this context, discharge readiness for parents is defined as “the masterful attainment of technical skills and knowledge, emotional comfort, and confidence with infant care at the time of discharge”, and discharge preparation is “the process of facilitating comfort and confidence as well as the acquisition of knowledge and skills to successfully transition home” [19]. International professional organisations have proposed key criteria to improve readiness for discharge: a) the assessment of maternal and infant physiological stability; (b) knowledge, ability, and confidence regarding self-care and infant care; (c) availability of support at home; and (d) availability of obstetric and infant care following discharge [11, 20].

It is unclear whether or how they are being used by healthcare providers in discharge preparation procedures with women, parents and families. We used these defintions to develop the inclusion criteria, inform the search strategy and to guide the subsequent summary of criteria in use in the included policy and research documents. Table 1 lists the inclusion criteria used to determine the documents to be included in the review.

**Table 1:** Inclusion and exclusion criteria applied in the scoping reveiw

	Inclusion criteria	Exclusion criteria
Participants	Women, newborns and parents/caregivers/family members post-birth in a facility Midwives/nurses/other health workers or providers of care in a facility prior to discharge after birth	Other participant groups, unrelated to postnatal care Women, newborns and parents/caregivers/family members after a home birth
Intervention	Discharge preparation or discharge readiness after facility birth	
Context	Any country	
Outcomes	Definitions of discharge preparation and discharge readiness Criteria and content for assessing readiness for discharge Description of interventions to improve discharge preparedness/discharge readiness	
Type of document	Policy documents, guidelines, consensus statements, protocols, job aids, tools/checklists Published research with any study design and other literature (e.g. conference abstracts, commentaries) Unpublished documents including technical reports and dissertations	
Language	No language restrictions although the search was conducted in English	
Date limits	From 2000 onwards, when studies on the effect of ‘postnatal discharge’ began to appear in the literature	Prior to 2000

### Search strategy

#### Policy and guideline retrieval

To identify existing guidelines, policies or professional consensus statements on discharge preparation and readiness we searched websites and archives of international and national organisations known to develop or archive guidelines: International Guideline Library of the Guidelines International Network, the Canadian Medical Association Infobase, National Institute for Health & Care Excellence (NICE), Agency for Healthcare Research and Quality, Institute for Clinical Systems Improvement, Institute for Health, and Institute for Healthcare Improvement. We also looked at websites and contacted individuals of organisations and professional societies working on postnatal care: United Nations Children’s Fund (UNICEF), United Nations Population Fund (UNFPA), World Health Organization (WHO), International Federation of Gynecology & Obstetrics (FIGO), European Board & College of Obstetricians and Gynaecologists, American College of Obstetrics & Gynecology, Royal College of Obstetrics & Gynaecology, Royal Australian and New Zealand College of Obstetricians and Gynaecologists, and the International Confederation of Midwives.

A general Google search was conducted to supplement the above using keywords relating to recommendations, guidelines and policies and discharge readiness.

#### Literature search

We followed the Joanna Briggs Institute recommended approach for scoping reviews to locate published research and other literature [17]. First, we listed terms and synonyms relevant to each of the inclusion criteria. These were reviewed by a WHO librarian. An initial search of a few relevant databases was performed, the text words used in retrieved article titles and abstracts were analysed, and then a comprehensive search of all relevant databases was conducted using all identified key words and index terms.

Fourteen electronic databases were searched using a multi-stage process. An initial high level search of multidisciplinary databases within EBSCO and ProQuest interfaces was conducted using main keywords in [title] only to find niche and/or rare papers. This was followed by separate searches of individual databases using their specific syntax (see Additional file 1 for an example): CINAHL, MEDLINE, PsychINFO, Scopus, Web of Science, Google Scholar, LILACS, EMBASE, AJOL, Global Health. Finally we searched Global Index Medicus (GIM) hosted by WHO, and African Index Medicus (AIM) using the (more up to date) native interface.

### Screening and selection

All articles, reports and documents retrieved from database, website and archive searches were saved in a Mendeley database. One author (HS) screened the titles and abstracts of all records; a second author (AGP) independently screened 25% of the records. The same author (HS) assessed relevant full text documents against the inclusion criteria and AGP independently assessed 25% of the full texts. Results were compared and discrepancies resolved by discussion and returning to the papers. At the full text screening, reasons for exclusion were recorded.

**Fig. 1:**
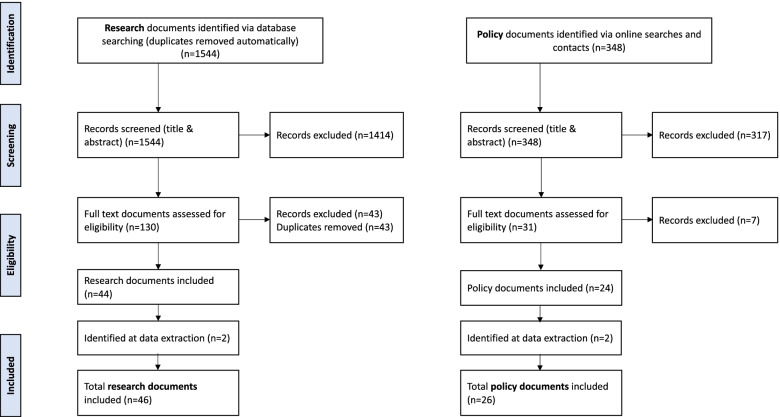
Flow diagram of screening and selection process

Screening and selection decisions are documented in the PRISMA flow chart (Fig. 1).

### Data extraction

Separate MS Excel spreadsheets were used to extract and chart information relevant to the review objectives from a) policy and guideline documents and b) research and other literature. A data extraction form was piloted to ensure relevance of all fields. One author (CH) extracted information and another (AGP) checked 20% of the documents.

### Summarising and reporting the findings

The following information was extracted for all included documents: key characteristics including year of publication, country of origin, area of intervention (discharge preparedness, discharge readiness); type of document; and where applicable, description of intervention implemented; and definitions of discharge preparation and discharge readiness provided.

To further summarise discharge readiness criteria or components, a second spreadsheet was created to list criteria and map these by source (policy documents and research and other literature). Using the minimum discharge criteria defined by the American Association of Pediatrics [21] as an initial framework, we then conducted a content analysis and inductively derived categories of discharge assessment criteria in use in the policy and research documents. A third separate spreadsheet was created to map discharge preparation steps and common content within each step, by source (policy documents and research and other literature).

Finally for those documents that described implementation or evaluation of interventions to deliver discharge preparation or readiness, the following information was characterized; a) the type of intervention, b) the study design, c) participants, d) the intervention content, e) the timing of intervention delivery and f) the otucomes measured. Other clusters of information were identified in the process of summarising the extracted information from each document. Research documents that reported qualitative research on experiences of postnatal discharge, theoretical or conceptual frameworks for discharge readiness, and scales for measuring or scoring discharge readiness were also summarised.

Reporting of the scoping review findings follows the PRISMA-ScR (Preferred Reporting Items for Systematic reviews and Meta-Analyses extension for Scoping Reviews) format [22].

## Findings

### Description of included documents

We identified 348 policy and guideline documents, of which 26 met the inclusion criteria (see Fig. 1). In addition, we identified a total of 1544 research or other literature documents from database searches, of which 46 met the inclusion criteria. One of the research documents is in Bulgarian; it has not been translated and is referenced but not cited in the report [23]. Additional file 2 includes a full list of included documents and their characteristics and Tables 2 and 3 summarise key characteristics.

**Table 2:** Characteristics of included policy and guideline documents

Characteristic	Number of documents (*N* = 26)
Type of document	
Guideline	12
Profesional statement	3
Checklist	3
Measurement scale	2
Clinical protocol	1
Handbook	1
Policy	1
Poster	1
Technical consultation	1
Toolkit	1
Country of origin	
USA	8
Global	7
UK	5
Canada	2
Spain	1
India	1
Iran	1
Northern Ireland	1
Discharge focus	
Readiness	9
Preparation	8
Both	5
Unclear	4
Orientation	
Postnatal	23
Generic	2
Pre-term infants	1

**Table 3:** Characteristics of included research and other literature

Characteristic	Number of documents (*N* = 45^a^)
Type of document	
Research article	35
Confernece abstract	2
Evaluation/report	2
Thesis	3
Commentary	2
Medical news article	1
Study design	
Cross-sectional/ descriptive/ correlational	11
Qualitative	6
Before and after/ pre-post/ non-randomised evaluation/ comparative	6
Propsective cohort	5
Quality improvement / knowledge translation	4
Review	4
Randomised controlled trial	3
Reliability study	1
Unclear	1
Not applicable^b^	4
Geographical location	
North America Canada (*n* = 2), USA (*n* = 15)	17
Europe England (*n* = 2), France (*n* = 3), Ireland (*n* = 1), Poland (*n* = 1), Spain (*n* = 3), Sweden (*n* = 2), Turkey (*n* = 6), UK (*n* = 1)	19
Middle East Iran (*n* = 1), Israel (*n* = 1), Jordan (*n* = 1), Lebanon (*n* = 1)	4
Africa Tanzania (*n* = 1)	1
Asia Taiwan (*n* = 1), Thailand (*n* = 1)	2
South America Brazil (*n* = 1), Venezuela (*n* = 1)	2
Discharge focus	
Readiness	22
Preparation	16
Both	6
Unclear	1
Orientation	
Postnatal (mother and newborn)	36
Pre-term infants	6
Hospitalised children	2
Generic	1

^a^*n* = 1 paper in Bulgarian not translated [23]

^b^two commentaries, one medical news article and one report

Just under half the policy and guideline documents were ‘guidelines’ (*n* = 12) and of these 10 were national and two global (Table 2). The three professional society statements were developed by the American College of Obstetricians & Gynecologists (*n* = 1) and the Canadian Paediatric Society (*n* = 1). The remainder of the documents were checklists (*n* = 3), scales to assess discharge readiness (*n* = 2), and one each of the following: a clinical protocol, handbook, poster, technical consultation and a toolkit. These are referred to as policy documents throughout. The majority of documents originated in the United States of America (USA) (*n* = 8) or were global (*n* = 7); the remainder were from the United Kingdom (UK) (*n* = 5), Canada (*n* = 2), Spain (*n* = 1), India (*n* = 1), Iran (*n* = 1) and Northern Ireland (*n* = 1).

The research and other literature included published research articles (*n* = 35), conference abstracts (*n* = 2), evaluations or reports (*n* = 2), theses (*n* = 3), commentaries (*n* = 2) and a medical news article (*n* = 1) (Table 3). These documents are referred to as ‘research documents’ throughout. Most research was conducted in Europe (England (*n* = 2), France (*n* = 3), Ireland (*n* = 1), Poland (*n* = 1), Spain (*n* = 3), Sweden (*n* = 2), Turkey (*n* = 6), UK (*n* = 1)) and North America (Canada (*n* = 2), USA (*n* = 15); the remainder in the Middle East (Iran (*n* = 1), Israel (*n* = 1), Jordan (*n* = 1), Lebanon (*n* = 1)), Africa (Tanzania (*n* = 1)), Asia (Taiwan (*n* = 1), Thailand (*n* = 1)) and South America (Brazil (*n* = 1), Venezuela (*n* = 1)). Most of the documents concerned postnatal discharge (*n* = 36); others concerned discharge of pre-term infants (*n* = 6), hospitalised children (*n* = 2) and one was a generic discharge programme. More documents focused on discharge readiness (*n* = 22) than discharge preparation (*n* = 16); six focused on both and in one the focus was unclear. The study designs of included research articles were largely descriptive (cross-sectional, correlational or descriptive (*n* = 11), before and after type studies (*n* = 6) or qualitative (*n* = 6). Other studies used prospective designs (*n* = 5), quality improvement or knowledge translation approaches (*n* = 4); three were randomised controlled trials, four were review articles and one a reliability study.

### Definitions of discharge preparation and readiness

We found few explicit definitions of discharge preparation or readiness in the research and policy documents. The only policy document that defined discharge preparation stated that it requires a systematic and multidisciplinary approach, that parents should have an active role and health care providers should ensure that the family achieves competencies during the transition to home [24]. Research documents offered loose defnitions of discharge preparation, most of which emphasised the ‘provision of education, information or instructions’ to mothers about taking care of the newborn and their own health after birth [14, 15, 25–30]. Some specifically referred to preparation for the ‘transition home’ or to adapt to ‘changes’ in the woman’s and newborn’s lives [12, 26, 27, 31, 32]. More recent research articles mentioned empowering parents or helping them take control and make their own decisions as an important step in discharge preparation [30, 31].

Policy documents offered defintions of discharge readiness that mentioned assessment of physical or medical readiness for discharge, but acknowledged that confidence of the mother, social risk factors, support available at home and access to follow up care were also important [24, 33, 34]. Defnitions in research documents recognised that the decision to discharge was ‘complex’ and varied depending on the confidence of the mother to take care of the baby at home, support and stability at home, access to follow up care, social vulnerabilities and psychological adaptation [30, 35, 36]. In three research documents defnitions mentioned ‘joint assessment ‘or ‘agreement’ between the mother, family and health professionals that both the mother and infant were ready for discharge [9, 10, 31].

### Mapping of discharge readiness criteria

Thirteen policy documents reported discharge readiness criteria [24, 33, 34, 37–46]. Seventeen research documents reported readiness criteria, and these included nine research studies [11, 12, 25, 31, 32, 35, 47–49], three review articles [36, 50, 51], two commentaries [52, 53], a thesis [54], a medical news article [55] and an unpublished evaluation report [56]. Policy documents were from the United States (*n* = 3), Canada (*n* = 2), India (*n* = 2), England (*n* = 1) and five of the policy documents had a global focus. Research documents came from the USA (*n* = 5), France (*n* = 3), Turkey (*n* = 2), England (*n* = 1), Poland (*n* = 1), Chile (*n* = 1), Spain (*n* = 1), Ireland (*n* = 1), Canada (*n* = 1) and Venezuela (*n* = 1). Table 4 shows the mapping of discharge readiness criteria contained in the included research and policy documents.

**Table 4:** Mapping of discharge readiness criteria in research and policy documents

Crtieria and domains	Research documents (*N* = 17) n [%]	Policy documents (*N* = 13) n [%]
Assessment of maternal and infant physiological stability	15 [88%] [11, 12, 25, 31, 32, 35, 36, 47, 49–51, 53, 55, 56]	12 [92%] [24, 33, 34, 37–39, 41–46]
Maternal health (e.g. physical exam, danger signs, pain/discomfort)	10 [59%] [11, 12, 31, 32, 47, 49, 50, 53, 55, 56]	7 [54%] [37, 39, 41–43, 45, 46]
Infant health (e.g. physical exam, danger signs, breastfeeding, vnutrition/weight status)	10 [59%] [32, 35, 36, 47, 50–53, 55, 56]	10 [77%] [24, 33, 34, 37, 41–43, 45, 46]
Low birth weight (e.g. stable breathing, full feeds, sustained weight gain)	1 [6%] [25]	1 [8%] [24]
Tests (e.g. maternal serologies, infant metabolic screening, infant hearing screening, infant APGAR test)	8 [47%] [36, 47, 50–53, 55, 56]	7 [54%] [24, 33, 34, 37, 38, 42, 44]
Treatment (e.g. newborn immunisations, infant HepB vaccine, ARVs for mother and baby if HIV+)	6 [35%] [32, 35, 36, 51, 52, 56]	8 [62%] [24, 33, 34, 37, 38, 42–44]
Knowledge, ability and confidence regarding self-care and infant care	13 [76%] [11, 12, 25, 31, 35, 36, 47–53]	11 [85%] [24, 33, 34, 37–40, 42–45]
Education (information provided to mother on various topics e.g. breastfeeding, care of newborn, car seat safety, family planning, hygiene practices)	3 [18%] [36, 47, 52]	10 [77%] [24, 34, 37–40, 42–45]
Low birth weight (e.g. training for parents on oxygen and/or tube feeds)	1 [6%] [25]	0
Assessment of maternal confidence and knowledge (e.g. breastfeeding, caring for baby, caring for self, identifying danger signs, infection control)	11 [65%] [11, 12, 31, 35, 47–53]	4 [31%] [24, 33, 34, 37]
Availability of support at home	15 [88%] [11, 12, 31, 32, 35, 36, 47–49, 51–56]	3 [23%] [24, 33, 34]
Home environment (e.g. family support, violence, financial concerns)	13 [76%] [11, 12, 31, 32, 35, 48, 49, 51–56]	3 [23%] [24, 33, 34]
Social risk factors (e.g. psychosocial concern, smoking, alcohol/substance use, language/access barriers to service)	9 [53%] [32, 35, 47, 51–56]	3 [23%] [24, 33, 34]
Availability of maternal and infant care following discharge	8 [47%] [32, 36, 47, 48, 50–53]	8 [62%] [24, 34, 37–39, 42, 43, 45]
Follow-up (e.g. instructions provided, timely follow-up arranged/identified, identification of medical facility in case of emergency)	8 [47%] [32, 36, 47, 48, 50–53]	8 [62%] [24, 34, 37–39, 42, 43, 45]
Support services available to family (e.g. family must have a primary care provider, link to community postnatal services, lactation support)	3 [18%] [25, 51, 52]	3 [23%] [34, 37, 42]
Other	6 [35%] [35, 50, 51, 54–56]	9 [69%] [33, 37–41, 43, 44, 46]
Timing of assessment (ie. ‘x’ hours after birth)	2 [12%] [52, 56]	2 [15%] [43, 46]
Other (e.g. type of birth, multiparous, birth certificate provided)	5 [29%] [50, 51, 54–56]	7 [54%] [33, 37–41, 44]

All four minimum discharge readiness criteria defined by the AAP [21] were reported in the policy and research documents. Nearly all policy (*n* = 12) and research (*n* = 15) documents mentioned assessment of maternal and infant physiological stability as a criterion. In policy and research documents components for assessing the condition of the newborn (physical examination of the newborn and nutrition and weight status of the newborn) were mentioned more often than assessment of maternal status.

Almost all policy (*n* = 11) and research documents (*n* = 13) reported assessment of knowledge, ability and confidence regarding self-care for the woman and infant care. Policy documents were more likely to report use of written educational materials (*n* = 10), commonly covering topics such as breastfeeding, care of the newborn, danger signs and family planning. Four policy documents mentioned assessment of aspects of maternal confidence and knowledge, including identification of danger signs and confidence in caring for the baby. The research documents were more likely to report components to do with assessing maternal confidence and knowledge (*n* = 11).

Policy (*n* = 8) and research (*n* = 8) documents mentioned assessment of availability of obstetric and infant care following discharge. Assessing timely follow-up arrangements was the most frequently reported component (policy documents *n* = 7; research documents *n* = 9). Other components mentioned for this criterion included identification of a medical facility in case of emergency (policy documents *n* = 3; research documents *n* = 4), family must have a general practitioner (research documents *n* = 4), link to community postnatal services (policy documents *n* = 3), follow up instructions or plan (policy documents *n* = 2; research documents *n* = 1), and immunisations arranged (policy documents *n* = 2).

Assessment of availability of support at home was much more frequently reported in research documents (*n* = 15) than policy documents (*n* = 3). Research documents mentioned a broad range of home environment factors considered important to assess at discharge including family support (*n* = 9), domestic violence (*n* = 2) and financial concerns (*n* = 4). Psychosocial concerns (*n* = 6), alcohol or substance misuse (*n* = 3), and social risk factors included language barriers (*n* = 4), local residence or access barriers to services (*n* = 2) and age of the mother (*n* = 2).

### Mapping of discharge preparation steps

Fifteen policy documents [2, 4, 24, 34, 37, 42, 45, 46, 57–63] and 11 research documents [14, 25–27, 36, 64–69] reported discharge preparation steps. Table 5 shows the mapping of discharge preparation steps contained in the included research and policy documents.

**Table 5:** Mapping of discharge preparation steps in research and policy documents

Discharge preparation steps	Research documents (*N* = 11) n [%]	Policy documents (*N* = 15) n [%]
Provide information to women and families on topics related to self-care of woman, care of newborn, danger signs, follow-up care and home, family and social support	9 [82%] [25–27, 64–69]	12 [80%] [2, 24, 34, 45, 46, 57, 58, 60–63]
Assess need or refer to services	5 [46%] [14, 36, 66–68]	12 [80%] [4, 24, 34, 37, 42, 46, 58–63]
Plan follow-up care	2 [18%] [66, 67]	5 [33%] [24, 34, 59, 62, 63]
Provide opportunity to talk about birth experience and ask questions about care received	0	4 [27%] [2, 4, 60, 62]
Complete home-based record for the woman and baby	0	3 [20%] [34, 60, 62]
Provide a discharge care plan	3 [27%] [14, 66, 67]	2 [13%] [59, 63]

Our mapping identified six commonly reported steps: a) provide information to women and families on a range of topics; b) assess need or refer to services; c) plan follow-up care; d) provide opportunity to talk about birth experience and ask questions about care received; e) complete home-based record for the woman and the baby; and f) provide a discharge care plan. Of these common steps, nearly all the policy (13/15) and research documents (9/11) reported a step in the process for providing information to women and their families. Topics for discharge education or information ranged from self-care for the woman, care of the newborn, advice on danger signs, home, family and social support, and follow-up care. Three research articles mentioned the format of information – mainly written materials or teaching delivered by discharge educators.

Few research documents reported on additional discharge preparation steps besides providing information: assess need or refer to services (*n* = 5); provide opportunity to talk about birth experience and ask questions about care received (*n* = 0); complete home-based record for the woman and the baby (*n* = 0); and provide a discharge care plan (*n* = 3); plan follow-up care (*n* = 2). Policy documents were more likely to report on additional steps in the discharge preparation process: assess need or refer to services (*n* = 12); plan follow-up care (*n* = 5); provide opportunity to talk about birth experience and ask questions about care received (*n* = 4); complete home-based record for the woman and the baby (*n* = 3); and provide a discharge care plan (*n* = 2).

### Interventions to improve delivery of discharge preparation

Nine research papers reported interventions to improve the delivery of discharge preparation in normal vaginal birth and in normal term infants (Table 6). The studies were mainly small-scale pilots or evaluations involving fewer than 250 participants and predominantly comparing delivery of education through provision of written materials and training sessions to routine care. The studies were conducted in Europe (Turkey *n* = 2), North America (Canada *n* = 1 and USA *n* = 3), South America (Brazil *n* = 1), Middle East (Israel *n* = 1, Lebanon *n* = 1). Seven studies included an education component or information provision for mothers, in varying formats. Written materials included a modified discharge letter [14], written booklets or brochures [64]. One study included a discharge folder and educational resources provide by a designated nurse [28]. Two studies implemented an education session before discharge [70, 71]; One study implemented a designated nurse and an educational material to provide discharge education to mothers [69]. One study reported a programme to enhance the discharge experience including interactive education and sensing sessions for women, adding emergency information to discharge instructions [29]; one study reported on a test of content through educational sessions using group dynamic activities prior to discharge [72] and one evaluated an innovative model of postnatal care to improve discharge preparation [56]. Two studies assessed the effectiveness of discharge education or information provision for mothers [64, 69] and reported effects on women’s satisfaction with care, postpartum visits to a health professional after discharge, and discharge readiness as measured on a scale. Other studies were descriptive (*n* = 2), cross-sectional (*n* = 3) or used quality improvement approaches (*n* = 2) and reported various outcomes including maternal recall of discharge intructions, maternal satisfaction with discharge procedure, and maternal readiness for discharge. Of those studies that reported on timing of intervention use, one was designed for use on admission [28], five at or around the time of discharge [14, 56, 64, 70, 71]. Two studies do not specify when the intervention is initiated [29, 69].

**Table 6:** Summary of intervention studies to strengthen discharge preparation

Country, author (year)	Aim	Study design	Participants	Orientation and description of intervention	Reported outcomes
Discharge preparation in normal birth and normal term infants (*n* = 9)					
Arad (2007) [14] Israel	To assess the maternal recall of the neonatal discharge letter instructions with and without nurse and mother signing the document in addition to the physician signature	Before and after	Mothers of infants born when nurse & mother signatures required (*n* = 109 Mothers of infants born when only physician signature required (*n* = 110)	Use of a neonatal discharge letter with instructions, changed to require nurse and mother signatures in addition to physician who examined the baby, to improve maternal recall of information delivered in the letter At discharge	Whether discharge letter was understood; Staff openness for questions at discharge; Maternal recall of discharge instructions; Maternal satisfaction with dicharge procedure
Dag 2013 [71] Turkey	To evaluate postpartum discharge readiness in women who had vaginal birth	Evaluation (cross-sectional survey)	Women who had normal vaginal delivery (*n* = 110)	No formal intervention in the study; women’s discharge readiness was assessed based on the routine care and education given at the time of discharge At discharge	Breastfeeding rate at 1 month; readmmission or consultation rate in 1st month; rate of missing first exam or screening tests at 8 days; and parents satsfaction with care
Fleischmann (2015) [29] USA	To enhance the discharge experience of the postpartum woman	Quality improvement (Six Sigma workout)	Not stated	Innovative program to enhance the discharge experience of postpartum women including: conducting sensing sessions with patients, adding additional emergency information to discharge instructions, querying other large women’s hospitals, teleconferencing with a top decile hospital, adding questions related to Help at Home video to the Get Well Network interactive woman education system, adding a Discharge Pathway to the Get Well Network, and performing a research study on discharge readiness Timing not stated	Hospital Consumer Assessment of Hospital Providers and Systems (HCAHPS) discharge domain question scores
Kabakian-Khasholian (2007) [64] Lebanon	To evaluate the impact of providing women with written educational material on their satisfaction with care, and use of health services postpartum	RCT	Women having a live birth Intervention (*n* = 187) Control (*n* = 191)	Providing women with written educational booklet that addressed health problems, breastfeeding, contraception, postpartum check-up, father’s role and women’s weight problems after pregnancy to improve satisfaction with care, and use of health services postpartum At discharge	Postpartum visit to a health professional after discharge and before follow-up interview Satisfaction with maternity care received during pregnancy, childbirth and postpartum
Meringer (2015) [28] USA	To improve patient perception of readiness for discharge, by modifying delivery of care	Quality improvement (post- implementation survey)	Mothers and babies (sample size not stated)	Blue discharge folder to be initiated upon admission and serve as a living document to record education and follow the woman throughout her stay. It serves as a reference for the interprofessional team as well as a continuous resource for home On admission	Maternal readiness for discharge.
Matozinhos (2011) [72] Brazil	To evaluate content and activities to be included in discharge orientaiton	Descriptive (pre and post test)	Postpartum women (*n* = 73) and and accompanying person (19) who had given birth in the previous 25 h for vaginal birth or 48 h for a caesarean section located in a maternity ward of a public health institution.	A discharge orientation was provided through a group education session, using three different group dynamic activitie. An individual and individual post-test were applied.	Perceptions of maternal self-care, breastfeeding and care of the newborn
Salvador (2020) [56] Canada	To describe the MPCH program model of care, maternal-newborn low-risk criteria and present the preliminary evaluation results of the program	Evaluation (cross- sectional survey)	Women who gave birth between Dec 2018-Dec 2019 and received care from the MPCH Program (*n* = 100)	Monfort Postnatal Care at Home Program (MPCHP) is an innovative alternative to the traditional hospital postnatal model which safely shifts early postnatal care for low-risk dyads from hospital to home 6-24 h after a vaginal birth / 24-48 h after caesarean birth	Postpartum hospital length of stay; Parental support required following hospital discharge; Breastfeeding at 6 weeks; Maternal satisfaction with MPCH programme
Türkmen (2017) [70] Turkey	To investigate status of discharge readiness after education	Evaluation (cross-sectional survey)	Women who had normal vaginal delivery: received training (*n* = 99) no training (*n* = 24)	Education session implemented by health care provider before discharge At discharge	Maternal readiness for discharge
Wilson (2016) [69] USA	To evaluate the effectiveness of a designated nurse providing discharge education to increase the postpartum women’s perception of readiness for discharge	Non-randomised evaluation	Postpartum women who gave birth to a healthy term newborn with no perinatal complications Intervention (*n* = 30) Control (*n* = 30)	A designated nurse providing discharge education to increase the postpartum women’s perception of readiness for discharge Focused on maternal perception of readiness but it is a discharge preparation intervention Timing not stated	Discharge preparedness (personal status knowledge, coping ability, expected support)
Discharge preparation starting at 32–36 weeks of pregnancy (*n* = 1)					
Altuntug (2013) [73] Turkey	To assess effect of education on discharge readiness, postpartum complaints and postpartum quality of life	Non-randomised evaluation	Pregnant women 32–36 weeks Intervention (*n* = 40) Control (*n* = 40)	Preparation Educational progam for pregnant women comprising 3 sessions: 1) at 32–36 weeks readiness for labour, birth and postpartum issues; 2) before discharge newborn care and breastfeeding; 3) 4–6 weeks post-birth education about self-efficacy and quality of life after childbirth 32–36 weeks of pregnancy; before discharge; 4–6 weeks after childbirth	Discharge preparedness and quality fo life
Discharge preparation for pre-term babies (*n* = 4)					
Ingram (2016) [25] England	To investigate whether introducing the parent-centred neonatal discharge package (Train-to-Home) increased parental confidence in caring for their preterm infant (self-efficacy), reduced infants’ length of hospital stay and reduced healthcare resource use after discharge	Before and after	Infants without major anomalies born at 27–33 weeks’ gestation and their parents (*n* = 245 families)	Parent-centred neonatal discharge package (Train-to-Home) to increase parental involvement and understanding of their preterm baby’s needs, comprising a train graphic and supporting care pathways to facilitate parent’s understanding of their baby’s progress and physiological maturation, combined with improved estimation of likely discharge date Intervention is used for discharge preparation but part of it includes criteria for discharge readiness On admission	Infant attendance at emergency department; Cost of emergency department attendance; Infant length of stay; Number of re-admissions or outpatient appointments before and after implementation; Perceived parental self-confidence in caring for infant
Moradi (2018) [65] Iran	To determine the effect of an empowerment program on maternal discharge preparation and infants’ length of hospital stay	RCT	Mothers of premature infants in NICU Intervention (*n* = 33) Control (*n* = 32)	Maternal empowerment program initiated at admission to NICU until neonatal discharge and even after discharge. Family-oriented nursing intervention On admission, during stay until discharge	Women’s satisfaction with care; Women’s discharge preparedness; Newborn health; Length of hospital stay
Shieh (2010) [68] Taiwan	To evaluate the effectiveness of structured discharge education on maternal confidence and knowledge and the growth of premature newborn	RCT	Mothers with premature babies with no congenital abnormalities Intervention (*n* = 29) Control (*n* = 30)	Structured discharge education for maternal confidence and knowledge and the growth of premature newborns using a 15-page brochure of caring for premature infants. Brochure contents included the explanation of follow-up examination at clinics, newborn screening, management of emergent situations, feeding, temperature measurement, bathing, oral hygiene and eye care, stool and urination, medication and vaccination. Mothers asked to demonstrate the skill of care individually, after introduction of the brochure Timing not stated	Maternal confidence; Maternal caring knowledge; Infant growth change (height, weight)
Wangruangsatid (2012) [74] Thailand	Transitional care programme for mothers of preterm babies	RCT	Women with preterm infants Intervention (n=) Control (n=	Transitional care programme including 1.5–2 h classroom teaching session (with an educational video and a question and answer session) for mothers four days prior to infant’s discharge	Newborn morbidity; Newborn growth (weight, length, head circumference); Mother’s transition score (based on perceived knowledge and care giving skills and acceptance of being mother to a premature infant); Length of hospital stay

Other types of intervention reported in our included studies were one non-randomised study assessing the effect of discharge education through sessions starting at 32–36 weeks of pregnancy until 4–6 weeks after childbirth compared with routine care among women with healthy infants [73], and four studies of interventions to improve the delivery of discharge preparation for low birth weight or preterm babies [25, 65, 68, 74].

### Stakeholder perspectives on postnatal discharge

Six studies reported the perspectives of women, fathers and midwives on postnatal discharge using qualitative research methods. The studies were conducted in England, Sweden, Tanzania, and USA and number of participants ranged from 12 to 324. Two papers reported specifically on experiences of first-time mothers and fathers [75, 76] and two on the experience of early hospital discharge [76, 77]. Midwives’ and student midwives’ experiences are included in two papers [15, 78]. A qualitative evidence synthesis of these studies will be reported separately.

### Conceptual frameworks and theories of discharge readiness

Five research documents included conceptual frameworks or theories to help contextualise and understand the concepts of discharge preparation and readiness [11, 27, 32, 75, 77]. The theories and models reported in research articles were used in different ways. In some papers existing theories were used to guide the research being conducted and conceptualise linkages between the study variables [11, 27]. These frameworks represent mid-range theories, concerned with highly contextualised systems and processes of discharge [11, 27, 32]. For example, transitions theory helps place discharge preparation and readiness in the broader context of a ‘transition’ from facility to home, stipulating what is required to ensure the transition is as safe and effective as possible [11, 27]. In two qualitative papers, theoretical models were developed based on empirical findings [75, 77]. The lack of prepardnness model [75] and the sense of security model [77] represent micro-level theories, that help explain individual level behaviours and actions in relation to discharge. They prompt consideration of women’s and partners/father’s experiences of childbirth and the postnatal period, and emphasise the importance of customised approaches to facilitate acquisition of the knowledge and skills parents need to care for themselves and the baby. One paper reported developing a model of key drivers of successful discharge to help inform development of a quality improvement programme [32].

### Scales for measuring or scoring discharge readiness

Eleven research documents reported on scales for assessing or scoring readiness for discharge. Study designs included a RCT (*n* = 1), prospective cohort studies (*n* = 2), a before and after (*n* = 1), descriptive correlational studies (*n* = 4), cross sectional (*n* = 2) and a reliability study (*n* = 1). The studies were conducted in Europe (Poland *n* = 1; Turkey *n* = 3)), North America (US *n* = 5), Middle East (Jordon *n* = 1) and Asia (Taiwan *n* = 1). The scales reported in these studies were used to assess discharge learning needs or the quality of discharge teaching (*n* = 5), conduct readiness for discharge assessments (*n* = 6), and to conduct readiness assessments to support infant discharge from neonatal intensive care (*n* = 3).

Some scales were newly developed and not yet tested for reliability and validity (e.g. the Perceived Learning Needs (PLN) scale [7], the Neonatal Discharge Assessment Tool (N-DAT) [35], the maternal confidence scale and the caring knowledge scale [68]. The most commonly reported scale in use was the Readiness for Hospital Discharge Scale (RHDS), which was originally developed and validated in the US [11, 12, 27, 30, 31, 49]. Several adaptations of the RHDS exist – for new mothers, parents and nurses – and it appears to be the most comprehensive scale in use for assessing discharge readiness. The 23-item scale measures more than perceptions of physical health and includes an holistic assessment of the woman and her circumstances including emotional and psychological wellbeing, and expected social support and support in the home environment.

## Discussion

This scoping review addresses key knowledge gaps around discharge preparation and readiness in facilities prior to discharge after birth. It provides a systematic map of criteria for discharge readiness in use and the common steps healthcare providers take in preparing women, parents and newborns for the transition home. The mapping also identified from the literature interventions that have been used to strengthen discharge preparation, theories and models that conceptualise discharge preparation, scales for measuring discharge readiness and qualitative studies on the perspectives of women, fathers and healthcare providers on postnatal discharge.

### Discharge readiness assessment

Our mapping of criteria for assessing discharge readiness showed that assessment of maternal and infant physiological stability is the predominant criteria for assessing discharge readiness. Physical examination of the newborn was more commonly mentioned while assessment of maternal condition appeared to be less mentioned. The research documents favoured broadening the criteria beyond physiological assessment, to include assessment of the skills and confidence of the woman to take care of herself and of parents, caregivers and family to take care of the newborn, and also assessment of women’s emotional wellbeing. The research literature also indicated the importance of assessing the home environment that may impact on the ability to provide care in the home and other social factors which may affect care-seeking.

Including these criteria in future guidance and tools would allow health providers with women and parents, caregivers and families to identify and manage problems before discharge, and to provide information tailored to individual and family needs prior to discharge from the facility after birth. Where there is a need for additional support, links to relevant follow-up care and community-based services can be established. Future research could usefully determine effective strategies for linking facility and community health workers to ensure continuity of care and follow-up visits for women and newborns identified as high-risk, and for helping health workers prioritise additional support to women, newborns and families after discharge who need additional support.

These additional discharge criteria would also help health providers distinguish those women who live far from the facility, face language barriers, do not have access to transport and little support at home – where providers need to ensure linkages to the system for follow-up care.

### Discharge preparation steps

Our mapping of preparation steps revealed an emphasis on education or instructional components, which are important to help women and parents acquire knowledge and skills for the transition from facility to home. However, education is just one step in a process of helping women and families transition home. Other important steps reported in the policy documents are: ensuring a plan for follow-up care and completion of a home-based record for the woman and the baby. This should empower women, parents and families with knowledge of what should happen at discharge, beyond assessing danger signs and clinical condition. Only four documents reported on providing a discharge care plan. We found insufficient information to suggest what should be included in a discharge care plan, when it should be started or how this should be organised. This should be the focus of future research.

Of the interventions identified to strengthen discharge preparation, most were concerned with education or information provision for mothers after normal birth. We found a single study focused on discharge preparation sessions starting in pregnancy and four on discharge preparation for mothers and parents of pre-term babies. The educational interventions reported were diverse and we did not find enough evidence from studies using robust study designs to determine which approach is most effective for preparing women, men/fathers/partners and families prior to discharge from the facility. Only one study of structured discharge education for mothers of preterm babies examined caring knowledge; no other included studies assessed retention of knowledge, skills developed or the effect of discharge interventions on the efficiency of the system, burden on health services and readmissions. Given that post-birth discharge education is widely used, further research is needed to evaluate effectiveness of different approaches, using common outcome measures relating to the woman and her partner or father of the baby as well as impact on delivery of care and the health system. In some high-income countries women participate in pregnancy and early parenting classes that include preparation for the postnatal period. Further research is needed to determine if starting discharge preparation during pregnancy is effective and the benefits to mother and baby are retained in the postnatal period.

### Additonal insights from the research literature

It is highly likely that the positive effects associated with adequate discharge preparation (e.g. enhanced wellbeing, confidence and experiences) will be valued by women, their partners, parents, and families. However the context and health service conditions will affect the extent to which different approaches can be delivered. The information we retrieved on perspectives of women, men and healthcare providers, that will be reported in a separate paper, suggests that lack of time due to staff shortages, lack of staff training and availability of information in different languages, financial or insurance constraints affecting the length of stay and societal norms affecting how postnatal care education is received may affect approaches to strengthen discharge preparation. Other evidence from a qualitative synthesis of women’s experiences of postnatal care (Sacks, et al: Factors that influence uptake of routine postnatal care: findings on women’s perspectives from a qualitative evidence synthesis, submitted) suggests that in some contexts there are staff shortages, a lack of basic resources and a lack of privacy in postnatal settings, all of which may impact on the capacity to provide adequate discharge preparation for women. The quality of discharge preparation can also be influenced by the person delivering the information and education (e.g. their experience and qualifications), the woman receiving it (e.g. parity, education level, type of birth, type of infant feeding) and the context (e.g. high-risk infants, low-income countries). Another systematic review of providers’ views and experiences of postnatal care [79] suggests lack of personnel and heavy workload constrained the availability and quality of services, including care around the time of discharge after childbirth. Providers perceived the need to build trustful, sensitive relationships with women, and to provide then with sufficient and timely information to women. The lack of continuity of care and common policies or guidelines across different cadres and levels of maternal newborn health services may limit the offer of consistent information and breastfeeding counselling.

The theories and models identified in the research documents provide a lens through which discharge preparation and readiness can be viewed more broadly than the focus on physiological health, which is how they generally are conceptualised in clinical and medical-focused checklists currently in use. This thinking may be useful in identifying where to strengthen existing discharge preparation and readiness processes, and inform the development of specific interventions tailored to components of the discharge transition from facility to home. Similarly, the scales for assessing or scoring readiness for discharge we identified, particularly the RHDS which includes an holistic assessment of the woman and her circumstances, could be implemented at facility level. Scales such as these also offer a valid way of measuring the impact of interventions designed to improve discharge readiness or monitoring the implementation of discharge preparation steps and readiness criteria.

### Limitations

Our search strategy for locating policy documents was probably not as reliable as our approach to finding research and other literature, so we may not have retrieved all relevant policies, guidelines or professional consensus statements on discharge preparation and readiness. However, most policy and research documents tended to refer to the same professional statement – the minimum discharge criteria for a healthy term newborn defined by the American Association of Pediatrics [21]. The statement has been updated by the AAP committee several times since the original was published in 1995; the most recent reaffirmation was in 2015 [80]. Therefore we do not think we missed any substantially different or updated criteria or recommendations for discharge. Lastly, we are aware that our search may not have identified all non-English language research and policy documents. We did search without a language filter and drew on our network to help translate those we did find - in French, Spanish, Portuguese, Swedish, Turkish and Thai. One paper published in Bulgarian [23] is cited but not included in the review because we could not get it translated.

## Conclusion

This scoping review improves understanding of the nature of discharge assessment and how women, parents and newborns are prepared for the transition from facility to home after birth. One way to strengthen this transition put forward in the research literature is to assess prior to discharge women’s and families’ needs and circumstances in a more holistic way. Not only would this help to provide individualised information and support, it would also help staff distinguish between women and newborns who may require additional support. Strengthening discharge preparation requires health workers with the skills to provide information and counselling tailored to individual and family needs, adequate time, resources and supervision, and systems that enable linkage of facility and community-based health workers and support them to provide continuity of care for women and newborns after discharge.

## Supplementary Information

**Additional file 1.** Example search strategy for PubMed/MEDLINE (adapted for other databases).

**Additional file 2.** Characteristics of included documents.

## Data Availability

The data extracted and summarised in this scoping review is available from the corresponding author on reasonable requrest.

## Abbreviations

AAP: American Association of Pediatrics
AIM: African Index Medicus
AJOL: African Journals OnLine
CINAHL: Cumulative Index to Nursing and Allied Health Literature
DHS: Demographic and Health Surveys
EBSCO: Elton B. Stephens Company
EMBASE: Excerpta Medica dataBASE
FIGO: International Federation of Gynecology and Obstetrics
GIM: Global Index Medicus
LILACS: Latin American and Caribbean Health Sciences Literature
MEDLINE: Medical Literature Analysis and Retrieval System Online
N-DAT: Neonatal Discharge Assessment Tool
NICE: National Institute for Health and Clinical Excellence
PLN: Perceived Learning Needs scale
PNC: Postnatal Care
PRISMA-SCr: Preferred Reporting Items for Systematic reviews And Meta-analyses – Scoping Reviews
PsycINFO: Psychological Information Database
RCT: Randomised Controlled Trial
RHDS: Readiness for Hospital Discharge Scale
UK: United Kingdom
UNFPA: United Nations Population Fund
UNICEF: United Nations International Children’s Emergency Fund
USA: United States of America
WHO: World Health Organization

## Footnotes

1 Note on terminology: Throughout this paper we have used the term women, men, parents/caregivers and families. We recognise that not all individuals who go through childbirth identify as female. We also recognise that different types of couples and families exist. Although the literature we were scoping largely represented these terms, we recognise that future literature and any updates of this work will need to expand these concepts.
