# Supplementary material for: Discharge preparation and readiness after birth: a scoping review of global policies, guidelines and literature
Source: BMC Pregnancy Childbirth. 2022 Apr 5;22:281. doi: 10.1186/s12884-022-04577-3 (PMC8985304; doi:10.1186/s12884-022-04577-3)
Supplement: Supplementary file 1 — Additional file 1. Example search strategy for PubMed/MEDLINE (adapted for other databases). [file 12884_2022_4577_MOESM1_ESM.docx]

**Additional file 1. Example search strategy for PubMed/MEDLINE (adapted for other databases)**

| Search string | Content |
| --- | --- |
| #1 | Woman [mh] OR mother [mh] OR maternal [mh] OR patient [mh] |
| #2 | Infant [mh] OR new-born [mh] OR [baby] |
| #3 | Caregiver OR family [mh] OR partner [mh] OR husband [mh] OR parent [mh] |
| #4 | Midwife [mh] OR nurse [mh] OR health care worker [mh] OR health care professional [mh] OR health worker [mh] OR health care provider [mh] OR physician [mh] OR paediatrician [mh] OR obstetrician [mh] |
| #5 | #1 OR #2 OR #3 OR #4 |
| #6 | Discharge preparation [tiab] OR discharge readiness [tiab] OR readiness for discharge [tiab] OR discharge process [tiab] OR discharge OR discharge transition [tiab] OR discharge plan [tiab] OR hospital discharge [tiab] OR patient discharge [tiab] |
| #7 | Postnatal [mh] OR postpartum [mh] OR after birth [tw] OR intrapartum [mh] OR childbirth [mh] OR maternity [mh] OR neonatal [mh] |
| #8 | #6 AND #6 AND #7 |
| #9 | Publication year limit 2000-2020 |
