# Supplementary material for: Discharge preparation and readiness after birth: a scoping review of global policies, guidelines and literature
Source: BMC Pregnancy Childbirth. 2022 Apr 5;22:281. doi: 10.1186/s12884-022-04577-3 (PMC8985304; doi:10.1186/s12884-022-04577-3)
Supplement: Supplementary file 2 — Additional file 2. Characteristics of included documents. [file 12884_2022_4577_MOESM2_ESM.docx]

**Additional file 2. Characteristics of included documents**

Policies, guidelines and professional society documents (n=26)

| Author (year) | Organisation | Type of document | Country of origin | Title | Discharge focus | Orientation |
| --- | --- | --- | --- | --- | --- | --- |
| ACOG 2018 | American College Obstetricians & Gynecologists (ACOG) | Professional statement | USA | Optimising postpartum care | Unclear | Postnatal |
| Alaska Native Medical Centre 2015 | Alaska Native Medical Centre | Guideline | USA | Guideline: early discharge, newborn | Readiness | Postnatal |
| Cater 2016 | James Paget University Hospitals NHS Foundation Trust | Guideline | UK | Trust guideline for the management of postnatal care: planning, information and discharge guideline | Both | Postnatal |
| Department of Health Minnesota 2018 | Department of Health Minnesota | Guideline | USA | Newborn screening <24 hour discharge guideline | Preparation | Postnatal |
| Elliot Hospital 2012 | Elliot Hospital New Hampshire | Checklist | USA | Perinatal ticket to discharge | Readiness | Postnatal |
| Evans 2014 | Academy of Breastfeeding Medicine (ABM) | Clinical protocol | USA | Guidelines for hospital discharge of the breastfeeding term newborn and mother: the going home protocol | Preparation | Postnatal |
| Government of India 2015 | Maternal Health Division, Ministry of Health & Family Welfare | Guideline | India | Dakshata Empowering providers for improved MNH care during institutional deliveries: operational guideline | Readiness | Postnatal |
| GPC group 2014 | Guias de Practica Clinica (CPG) Ministerio de sanidad, servicios sociales e igualdad | Guideline | Spain | Clinical practice guidelines for care in pregnancy and the puerperium | Unclear | Postnatal |
| Henning 2015 | Southern Health and Social Care Trust | Guideline | N Ireland | Discharge newborn examination guideline for midwives | Readiness | Postnatal |
| Jack 2013 | Boston University | Toolkit | USA | Re-engineered discharge toolkit | Preparation | Generic |
| Jefferies 2014 | Canadian Paediatric Society | Professional statement | Canada | Going home; facilitating discharge of the pre-term infant | Both | Pre-term infants |
| Lemyre 2018 | Canadian Paediatric Society | Professional statement | Canada | Facilitating discharge from hospital of the healthy term infant | Both | Postnatal |
| NICE 2013 | National Institute for Health & Care Excellence (NICE) | Guideline | UK | Quality standard for PNC | Unclear | Postnatal |
| NICE 2015 | National Institute for Health & Care Excellence (NICE) | Guideline | UK | Postnatal care up to 8 weeks after birth | Preparation | Postnatal |
| Nolan 2018 | Nottingham University Hospitals NHS Trust | Policy | UK | Discharge policy for Greater Nottingham | Preparation | Generic |
| Pidgeon 2015 | Nottingham University Hospitals NHS Trust | Guideline | UK | Guideline for postnatal care | Preparation | Postnatal |
| Safe Motherhood National Program n.d. | Safe Motherhood National Program | Guideline | Iran | Integrated maternal health care: out of hospital services guideline | Unclear | Postnatal |
| USAID 2018a | United States Agency for International Development (USAID) | Checklist | Global | Postnatal care pre-discharge checklist | Readiness | Postnatal |
| USAID 2018b | United States Agency for International Development (USAID) | Poster | Global | Before discharge every mother and newborn needs counselling and care | Readiness | Postnatal |
| Weiss n.d. | Marquette University | Scale | USA | Readiness for Hospital Discharge Scale (RHDS): postpartum form | Readiness | Postnatal |
| Weiss n.d. | Marquette University | Scale | USA | Readiness for Hospital Discharge Scale (RHDS): registered nurse form | Readiness | Postnatal |
| WHO 2010 | World Health Organization (WHO)/Making Pregnancy Safer | Technical consultation | Global | WHO Technical Consultation on postpartum and postnatal care | Preparation | Postnatal |
| WHO 2013 | WHO/Maternal, Newborn, Child & Adolescent Health | Handbook | Global | A handbook for building skills: counselling for maternal and newborn healthcare | Preparation | Postnatal |
| WHO 2015a | WHO/Reproductive Health & Research | Guideline | Global | Pregnancy, childbirth, postpartum and newborn care a guide for essential practice | Both | Postnatal |
| WHO 2015b | WHO | Checklist | Global | WHO Safe Childbirth Checklist: implementation guide | Readiness | Postnatal |
| WHO 2018 | WHO/ Reproductive Health & Research | Guideline | Global | WHO recommendations: Intrapartum care for a positive childbirth experience | Both | Postnatal |

Research and other literature (n=45)*

| Author (year) | Type of document | Country of origin | Title | Discharge focus | Orientation | Study design |
| --- | --- | --- | --- | --- | --- | --- |
| Aguila 2000 | Research article | Spain | Registration of the newborn of normal term, scientific, administrative and ethical considerations | Readiness | Postnatal | Review |
| Almalik 2017 | Research article | Jordan | Understanding maternal postpartum needs: a descriptive survey of current maternal health services | Unclear | Postnatal | Prospective cohort |
| Altuntug 2013 | Research article | Turkey | Effects of health education on mothers’ readiness for postpartum discharge from hospital, on postpartum complaints, and quality of life | Readiness | Postnatal | Non-randomised |
| Arad 2007 | Research article | Israel | The impact of nurses and mothers signing the discharge letter on maternal knowledge and satisfaction after discharge from a neonatal unit: a before and after study | Preparation | Postnatal | Before and after |
| Arora 2018 | Research article | USA | Adherence to AAP healthy newborn discharge criteria in a tertiary care children’s hospital | Readiness | Postnatal | Cross-sectional |
| Bernstein 2002 | Research article | USA | Postpartum discharge: do varying perceptions of readiness impact health outcomes? | Readiness | Postnatal | Prospective cohort |
| Bernstein 2013 | Research article | USA | Unreadiness for postpartum discharge following healthy term pregnancy: impact on health care use and outcomes | Readiness | Postnatal | Prospective cohort |
| Bick 2011 | Research article | UK | Improving inpatient postnatal services: midwives views and perspectives of engagement in a quality improvement initiative | Preparation | Postnatal | Quality improvement (survey) |
| Buck 2020 | Research article | USA | Predictors of parenting readiness in fathers of high-risk infants in the Neonatal Intensive Care Unit | Readiness | Pre-term | Prospective cohort |
| Chanot 2009 | Research article | France | Home hospitalisation after early discharge from maternity hospital | Readiness | Postnatal | Prospective cohort |
| Dag 2013 | Research article | Turkey | Postpartum discharge readiness situation of women who had vaginal deliveries | Readiness | Postnatal | Evaluation (cross-sectional) |
| Dol 2019 | Research article | Tanzania | Exploring maternal postnatal newborm care and postnatal discharge education in Dar es Salaam, Tanzania: barriers, facilitators and opportunities | Preparation | Postnatal | Qualitative |
| Fleischmann 2015 | Conference abstract | USA | Improving women’s readiness for discharge preparation | Both | Postnatal | Quality improvement (Six Sigma) |
| Furzan (2007) | Research article | Venezuela | Early discharge of the newborn | Readiness | Postnatal | Review |
| George 2005 | Research article | USA | Lack of preparedness: experiences of first time mothers | Preparation | Postnatal | Qualitative |
| Girgin 2016 | Research article | Turkey | Validity and reliability of the Neonatal Discharge Assessment Tool | Readiness | Pre-term infants | Descriptive |
| Gracia 2017 | Commentary | Spain | Criteria for hospital discharge of the healthy term newborn after delivery | Readiness | Postnatal | n/a |
| Haith-Cooper 2018 | Research article | England | Hospital postnatal discharge and sepsis advice: perspectives of women and midwifery students | Preparation | Postnatal | Qualitative |
| Hascoet 2014 | Commentary | France | Maternity discharge: conditions and organisation for mothers and newborns return home. French National Authority for Health recommendations update | Preparation | Postnatal | n/a |
| Herrero-Morin 2015 | Research article | Spain | Maternal assessment of recommendations on the newborn infant care upon hospital discharge | Preparation | Postnatal | Cross-sectional |
| Ingram 2016 | Research article | England | Does family-centred discharge planning reduce healthcare usage? A before and after study in South West England | Both | Pre-term infants | Before and after |
| Jing 2017 | Research article | USA | Assessing maternal readiness for newborn discharge | Readiness | Postnatal | Review |
| Kabakian-Khasholian 2007 | Research article | Lebanon | Impact of written information on women’s use of postpartum services: a randomised controlled trial | Preparation | Postnatal | RCT |
| Kanotra 2007 | Research article | USA | Challenges faced by new mothers in the early postpartum period: an analysis of comment data from the 2000 pregnancy risk assessment monitoring system (PRAMS) survey | Preparation | Postnatal | Qualitative analysis of free-text data |
| Kaya-Senol 2017 | Research article | Turkey | An investigation of postpartum mothers’ readiness for hospital discharge and the affecting factors | Readiness | Postnatal | Cross-sectional |
| Laurent-Chevalier 2005 | Thesis | France | Early discharge from maternity, pediatric side. Evaluation of the home hospitalization service set up in Gérardmer in partnership with the Remiremont maternity hospital: retrospective study over 2 years | Readiness | Postnatal | Cross-sectional |
| Malagon-Maldonado 2017 | Research article | USA | Predictors of readiness for hospital discharge after birth: building evidence for practice | Both | Postnatal | Descriptive correlational |
| Matozinhos 2011 | Research article | Brazil | Evaluation of hospital discharge guidelines and its application to puerperal women sharing a room in a public hospital in Belo Horizonte | Preparation | Postnatal | Descriptive (pre and post test) |
| McEvoy 2009 | Medical news article | Ireland | Improving the safety of early newborn hospital discharge | Readiness | Postnatal | n/a |
| Meringer 2015 | Conference abstract | USA | Postpartum unit modifies delivery of care to enhance readiness | Readiness | Postnatal | Quality improvement (post- implementation survey) |
| Moradi 2008 | Research article | Iran | The effect of empowerment program on maternal discharge preparation and neonatal length of hospital stay: a randomised controlled trial | Preparation | Pre-term infants | RCT |
| Nagorska 2019 | Research article | Poland | The adaptation of Polish version of the Readiness for Hospital Discharge Scale (RHDS) for postpartum mothers | Readiness | Postnatal | Reliability study |
| Nova Scotia Reproductive Care Program 2002 | Report | Canada | Healthy babies, health families: postpartum and postnatal guidelines | Both | Postnatal | n/a |
| Persson 2002 | Research article | Sweden | Patients’ experience of early discharge from hospital after birth in Sweden | Readiness | Postnatal | Qualitative |
| Roberts 2019 | Research article | USA | Improving discharge planning using the re-engineered discharge programme | Preparation | Generic | Pre-post test |
| Salvador 2020 | Evaluation report | Canada | Montfort’s postnatal care program (MPCH Program) an innovative model shifting postpartum care from hospital to home | Readiness | Postnatal | Evaluation (cross-sectional) |
| Shieh 2010 | Research article | Taiwan | The effectiveness of structured discharge education on maternal confidence, caring knowledge and growth of premature newborns | Preparation | Pre-term infants | RCT |
| Svensson 2018 | Thesis | Sweden | First time mothers’ experiences of support from maternity ward related to early hospital | Preparation | Postnatal | Qualitative |
| Turkmen 2017 | Research article | Turkey | Examination of status of readiness for hospital discharge among puerperant women who gave vaginal delivery | Readiness | Postnatal | Evaluation (cross-sectional) |
| Tyler 2014 | Research article | USA | Development of a discharge readiness report within the electronic health record – a discharge planning tool | Both | Hospitalised children | Quality improvement (descriptive) |
| Weiss 2009 | Research article | USA | Predictors and outcomes of postpartum mothers’ perceptions of readiness for discharge after birth | Readiness | Postnatal | Descriptive correlational |
| Weiss 2017 | Research article | USA | Discharge teaching, readiness for discharge and post-discharge outcomes in parents of hospitalised children | Both | Hospitalised children | Descriptive correlational (secondary analysis) |
| Wilson 2016 | Thesis | USA | The effectiveness of the nurse discharge educator on postpartum women’s perception of readiness for discharge | Preparation | Postnatal | Non-randomised evaluation |
| Yanikkerem 2018 | Research article | Turkey | Factors affecting readiness for discharge and perceived social support after childbirth | Readiness | Postnatal | Cross-sectional |
| Wangruangsatid 2012 | Research article | Thailand | Effects of a transitional care programme for mothers of preterm babies | Preparation | Pre-term infants | RCT |

*n=1 paper in Bulgarian identified but not translated (Laleva, 2016)
